# The tip of the iceberg: challenges of accessing hospital electronic health record data for biological data mining

**DOI:** 10.1186/s13040-016-0109-1

**Published:** 2016-09-22

**Authors:** Spiros C. Denaxas, Folkert W. Asselbergs, Jason H. Moore

**Affiliations:** 1 Institute of Health Informatics, University College London, London, UK; 2 Farr Institute of Health Informatics Research, University College London, London, UK; 3 Department of Cardiology, Division Heart and Lungs, University Medical Centre Utrecht, Utrecht, Netherlands; 4 Institute for Biomedical Informatics, Department of Biostatistics and Epidemiology, Perelman School or Medicine, University of Pennsylvania, Philadelphia, PA 19104-6116 USA

## Abstract

Modern cohort studies include self-reported measures on disease, behavior and lifestyle, sensor-based observations from mobile phones and wearables, and rich -omics data. Follow-up is often achieved through electronic health record (EHR) linkages across primary and secondary healthcare providers. Historically however, researchers typically only get to see the tip of the iceberg: coded administrative data relating to healthcare claims which mainly record billable diagnoses and procedures. The rich data generated during the clinical pathway remain submerged and inaccessible. While some institutions and initiatives have made good progress in unlocking such deep phenotypic data within their institutional realms, access at scale still remains challenging. Here we outline and discuss the main technical and social challenges associated with accessing these data for data mining and hauling the entire iceberg.

In January 2015, President Barack Obama launched the Precision Medicine Initiative [1], a $215-million investment aiming to facilitate data-driven precision research by forging a cohort of at least one million participants. Primary data collection includes self-reported measures on disease, behavior and lifestyle, sensor-based observations from mobile phones and wearables, and rich -omics data. Follow-up will be achieved through electronic health record (EHR) linkages across primary and secondary healthcare providers. Historically however, researchers typically only get to see the tip of the iceberg: coded administrative data relating to healthcare claims which mainly record billable diagnoses and procedures. The rich data generated during the clinical pathway [2] (e.g. laboratory measurements, investigations, clinical notes, imaging, medications) remain submerged and inaccessible. While some institutions and initiatives [3–6] have made good progress in unlocking such deep phenotypic data within their institutional realms, access at scale still remains challenging. Here we outline and discuss the main technical and social challenges associated with accessing these data for data mining and hauling the entire iceberg.

It is often said that the field of informatics consists of people and technology intertwined. It comes as no big surprise that the greatest challenges are observed around interacting with clinical informatics staff and information systems. Research is usually not directly within the remit of informatics departments whose primary role is to support patient care through the provision and maintenance of various platforms and systems. This provision substantially varies between healthcare providers and across clinical specialties: providers might use a single unified EHR platform (e.g. Cerner, Epic) or a set of isolated platforms and systems integrated through bespoke middleware solutions. Often, these systems have been developed by subcontracted external software vendors which leads to substantial interaction costs when attempting to access data outside the standard clinical care use. In both cases however, it is usually the case that access to data for research has not been a key requirement and as a result the deployed platforms critically lack the functionality to facilitate it out of the box.

While the majority of secondary care clinical specialties generate electronic data, the manner in which data get captured and the context under which they are recorded differs. This results in a heterogeneous ecology of healthcare process models that even within a single provider are challenging to identify, integrate and re-use. It is often hard to get the “big picture” and discover the data flows between clinical departments and systems. The irregular utilization of metadata and health data standards makes it challenging to establish data provenance and assess data quality in a meaningful manner. More importantly, given the complexity of healthcare provision, it is difficult to establish the context under which data were generated and which is essentially required to enable the reuse of data for research. For example, the same piece of information, such as a blood pressure measurement or a white blood cell count, can be recorded across multiple systems but at differing temporal and clinical resolutions and in different contexts [7, 8].

Large amounts of information are also often stored in semi-structured or unstructured format. Biochemistry, haematology, microbiology and cellular pathology investigations and results are usually stored as semi-structured reports whose format varies significantly both within and between healthcare providers [9]. In some clinical specialties, such as mental health, the majority of information generated and recorded during interactions with clinical staff is stored as free-text [10]. Unstructured data are increasingly hard to access for research purposes and scalable natural language processing methods [11] and pipelines [12] are required in order to extract, clean and format these data at scale. Developing these tools however is equally difficult as access to large corpora of text which are required for algorithm training is restricted.

Data generated during clinical care are almost exclusively from unconsented patients which leads to ethical and governance challenges [13]. The reuse of such data for research requires a set of complex approvals from multiple governing entities which are challenging to navigate and obtain and operate in an opaque manner. Furthermore, significant concerns are often raised in terms of information security patient confidentiality and minimizing the risk of re-identification [14]. Researchers find themselves between a rock and a hard place. Research-driven environments offer substantially more flexibility in terms of analyzing the data such as for example through the provision of high performance clusters or flexible technology stacks that enable the development and evaluation of novel computational methods and approaches. At the same time, they are considered poorly in terms of information security and governance from healthcare providers who are reluctant to release data for storage there in large numbers or at high fidelity. Researchers often need to choose between working with a limited subset of the data in their own environment or with richer data in restrictive settings that directly hinder their productivity.

The challenges highlighted here underline the urgent need for new clinical informatics tools, theories and approaches in order to bridge the gap between the clinical care and research strata and accelerate the full translational continuum from basic research, to clinical trials and evaluation and integrated provision of healthcare at a population level [15, 16]. The complex and interdependent relationships that are observed between staff, platforms and data pose significant challenges for accessing data for research (e.g. in terms of cost or obtaining contextual knowledge) and performing research within hospitals (e.g. deploying a clinical decision support tool or undertaking integrated pragmatic clinical trials [17, 18]). Meaningful and sustainable relationships with clinical informatics staff need to be developed and nurtured in order to facilitate the bidirectional flow of knowledge. Furthermore, research should inform the requirements of such complex systems early on, enabling the scalable collection and curation of data in a transparent manner early on. Data mining is the key to insights from clinical big data but the data need to accessible and contain the information needed to improve healthcare.
